# Cross-Canada Variability in Blood Donor SARS-CoV-2 Seroprevalence by Social Determinants of Health

**DOI:** 10.1128/spectrum.03356-22

**Published:** 2023-01-10

**Authors:** Sheila F. O’Brien, Niamh Caffrey, Qi-Long Yi, Shelly Bolotin, Naveed Z. Janjua, Mawuena Binka, Caroline Quach Thanh, Derek R. Stein, Amanda Lang, Amy Colquhoun, Chantale Pambrun, Cassandra N. Reedman, Steven J. Drews

**Affiliations:** a Epidemiology and Surveillance, Canadian Blood Services, Ottawa, Ontario, Canada; b School of Epidemiology and Public Health, University of Ottawa, Ottawa, Ontario, Canada; c Center for Vaccine Preventable Disease, University of Toronto, Toronto, Ontario, Canada; d Dalla Lana School of Public Health, University of Toronto, Toronto, Ontario, Canada; e Department of Laboratory Medicine and Pathobiology, University of Toronto, Toronto, Ontario, Canada; f Public Health Ontario, Toronto, Ontario, Canada; g BC Centre for Disease Control, British Columbia, Vancouver, Canada; h School of Population and Public Health, University of British Columbia, British Columbia, Vancouver, Canada; i Department of Microbiology, Infectious Diseases & Immunology, Faculty of Medicine, University of Montreal, Montreal, Quebec, Canada; j Infection Prevention & Control, Clinical Department of Laboratory Medicine, CHU Sainte-Justine, Montreal, Quebec, Canada; k Cadham Provincial Laboratory, Winnipeg, Manitoba, Canada; l Faculty of Health Sciences, University of Manitoba, Winnipeg, Manitoba, Canada; m Roy Romanow Provincial laboratory, Saskatchewan Health Authority, Regina, Saskatchewan, Canada; n Population Health Assessment, Alberta Health, Edmonton, Alberta, Canada; o School of Public Health, University of Alberta, Edmonton, Alberta, Canada; p Medical Affairs & Innovation, Canadian Blood Services, Ottawa, Ontario, Canada; q Department of Pathology & Laboratory Medicine, University of Ottawa, Ottawa, Ontario, Canada; r Public Health Agency of Canada, Ottawa, Ontario, Canada; s Medical Microbiology Department, Canadian Blood Services, Edmonton, Alberta, Canada; t Department of Laboratory Medicine & Pathology, Division of Diagnostic and Applied Microbiology, University of Alberta, Edmonton, Alberta, Canada; Barcelona Centre for International Health Research (CRESIB, Hospital Clínic-Universitat de Barcelona)

**Keywords:** SARS-CoV-2, seroprevalence, Canada, blood donors, social determinants of health, racialization

## Abstract

We compared the seroprevalence of SARS-CoV-2 anti-nucleocapsid antibodies in blood donors across Canadian regions in 2021. The seroprevalence was the highest in Alberta and the Prairies, and it was so low in Atlantic Canada that few correlates were observed. Being male and of young age were predictive of seropositivity. Racialization was associated with higher seroprevalence in British Columbia and Ontario but not in Alberta and the Prairies. Living in a materially deprived neighborhood predicted higher seroprevalence, but it was more linear across quintiles in Alberta and the Prairies, whereas in British Columbia and Ontario, the most affluent 60% were similarly low and the most deprived 40% similarly elevated. Living in a more socially deprived neighborhood (more single individuals and one parent families) was associated with lower seroprevalence in British Columbia and Ontario but not in Alberta and the Prairies. These data show striking variability in SARS-CoV-2 seroprevalence across regions by social determinants of health.

**IMPORTANCE** Canadian blood donors are a healthy adult population that shows clear disparities associated with racialization and material deprivation. This underscores the pervasiveness of the socioeconomic gradient on SARS-CoV-2 infections in Canada. We identify regional differences in the relationship between SARS-CoV-2 seroprevalence and social determinants of health. Cross-Canada studies, such as ours, are rare because health information is under provincial jurisdiction and is not available in sufficient detail in national data sets, whereas other national seroprevalence studies have insufficient sample sizes for regional comparisons. Ours is the largest seroprevalence study in Canada. An important strength of our study is the interpretation input from a public health team that represented multiple Canadian provinces. Our blood donor seroprevalence study has informed Canadian public health policy at national and provincial levels since the start of the SARS-CoV-2 pandemic.

## INTRODUCTION

Early in the coronavirus disease 2019 (COVID-19) pandemic, blood donor severe acute respiratory syndrome coronavirus 2 (SARS-CoV-2) seroprevalence studies were implemented around the world to inform public health policy (1–6). Unlike routine COVID-19 case reports and contact testing (7, 8), seroprevalence studies estimate the population prevalence, independent of symptoms or public health testing constraints (3, 9). Canadian Blood Services has tested residual blood samples for SARS-CoV-2 antibodies each month since May 2020 in collaboration with the federal government COVID-19 Immunity Task Force (https://www.covid19immunitytaskforce.ca/). Blood donors are a healthy population and are not fully representative of the general population, but with blood collections in 9 of 10 provinces in all larger cities and most smaller urban areas, our study is the largest Canadian serosurvey.

Social determinants of health refer to nonmedical factors that influence the health of individuals and communities (10). Over the COVID-19 pandemic, cases have been more common in racialized (individuals who racially, ethnically, or culturally feel separated from the dominant culture) and socio-economically disadvantaged individuals, as well as in younger people, in many countries (11–14). Demographic and social determinants of health are used to address health inequity by targeting risk mitigation strategies and by allocating resources to underserved individuals and populations. COVID-19 studies are frequently focused on public health reported cases, which are biased by the availability of testing, the tenacity of contact tracing, and symptomatic infections.

Most Canadian studies have focused on particular regions and populations (15–19). Cross-Canada studies are rare because health information is under provincial jurisdiction and is not available in sufficient detail in national data sets, and other national seroprevalence studies have insufficient sample sizes for regional comparisons (20–22). A study of public health cases in four provinces identified higher cases in neighborhoods with more racialized individuals, a higher population density, a lower attainment of education, and a lower income (14). Higher age-standardized COVID-19 mortality was reported in ethno-cultural neighborhoods across Canada (23). Our blood donor data have the advantage of being near-national in reach, with consistent sampling and testing methodology. We have previously reported that racialized donors as well as material and social deprivation were independent predictors of SARS-CoV-2 seropositivity, but these associations have not been analyzed at the regional level (24). We aimed to compare the associations between socio-demographic variables and SARS-CoV-2 infection antibodies in blood donors in geographic regions across Canada in 2021.

## RESULTS

The number of donors and donations tested for each variable are shown in Table S1. There were 118,998 donors, from whom 165,236 donations were tested. About 40% of the donors and donations were from Ontario, and the next highest were Alberta (21%) and British Columbia (BC) (17%). The Prairies region (Saskatchewan and Manitoba) and the Atlantic region (New Brunswick, Nova Scotia, Prince Edward Island, and Newfoundland) each accounted for about 11% of the donations. A total of 139,150 donations (84%) were made in provinces from Ontario and westward, with 33 of 62 (50%) of the health regions having at least 1,000 tested. There were slightly more male donors than female donors (53% versus 47%), and donations were distributed across all age groups (17 to 24 years [9%], 25 to 39 [27%], 40 to 59 [36%], 60 and older [28%]). Donors were evenly distributed over social deprivation quintiles, but for material deprivation, they were overrepresented in the highest quintile and underrepresented in the two lower quintiles. About 17% of donors self-identified as being of a nonwhite race-ethnicity (including indigenous). The breakdowns of these racialized donors by race-ethnicity in each region are shown in Fig. S2. There percentage of racialized donors by region ranged from 25% in BC, 16% in Alberta, 18% in Ontario, and 15% in the Prairies to 6% in Atlantic Canada.

The proportion of anti-SARS-CoV-2 N antibody-positive donations varied across the country, with the highest proportions coming from Alberta (6.9%) and the Prairies regions (5.4%). These were followed by those from Ontario (3.7%) and British Columbia (3.7%), and there was low seroprevalence in the Atlantic region (0.5%) (Table S2).

**FIG 1:**
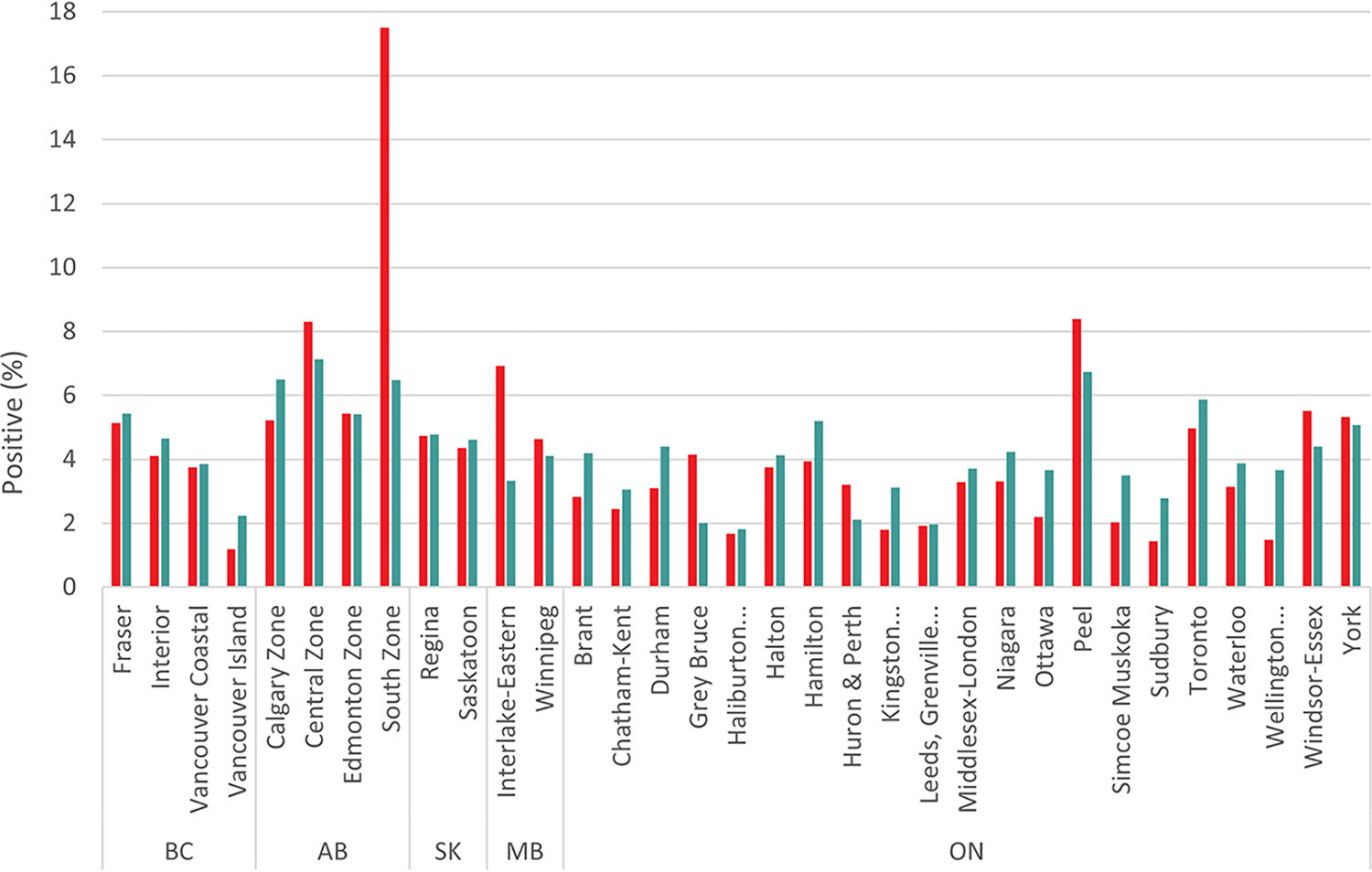
Percentage of SARS-CoV-2 PCR positive general population and seropositive donations by provincial health region (*r* = 0.45, *P* < 0.0001). Note: includes only health regions for which at least 1,000 donations were tested.

The seroprevalence in donors was correlated with the percentage of SARS-CoV-2 PCR positive public health cases by region (Fig. 1) (*r* = 0.45, *P* < 0.001). The Southern Zone of Alberta, which comprises southeastern Alberta, had markedly higher anti-SARS-CoV-2-N seroprevalence than cases. Many of these anti-SARS-CoV-2-N seropositive donors were living in rural areas of the province.

**FIG 2:**
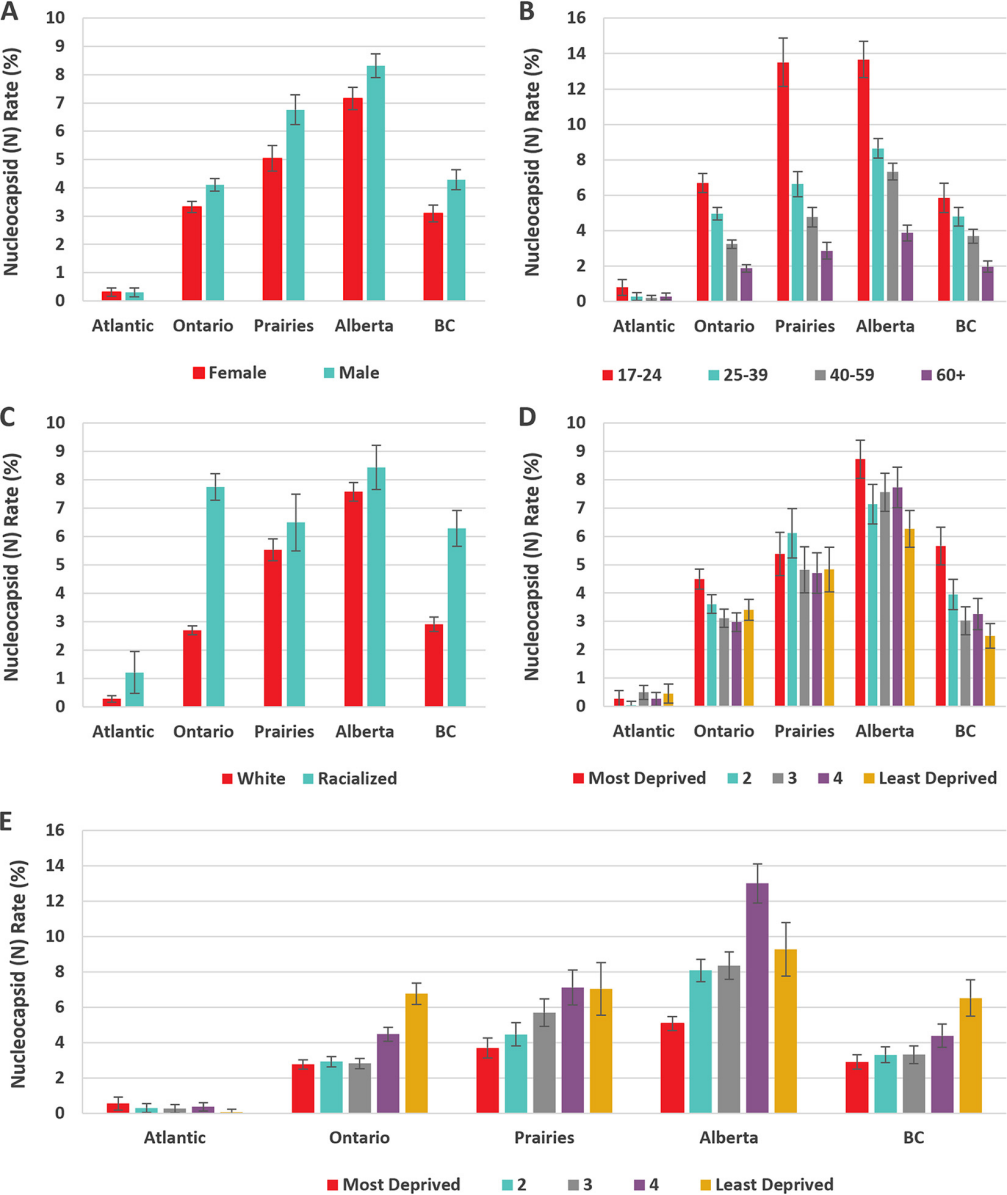
Regional seroprevalence by (A) sex, (B) age group, (C) racialization, (D) social deprivation quintiles, and (E) material deprivation quintiles.

**FIG 3:**
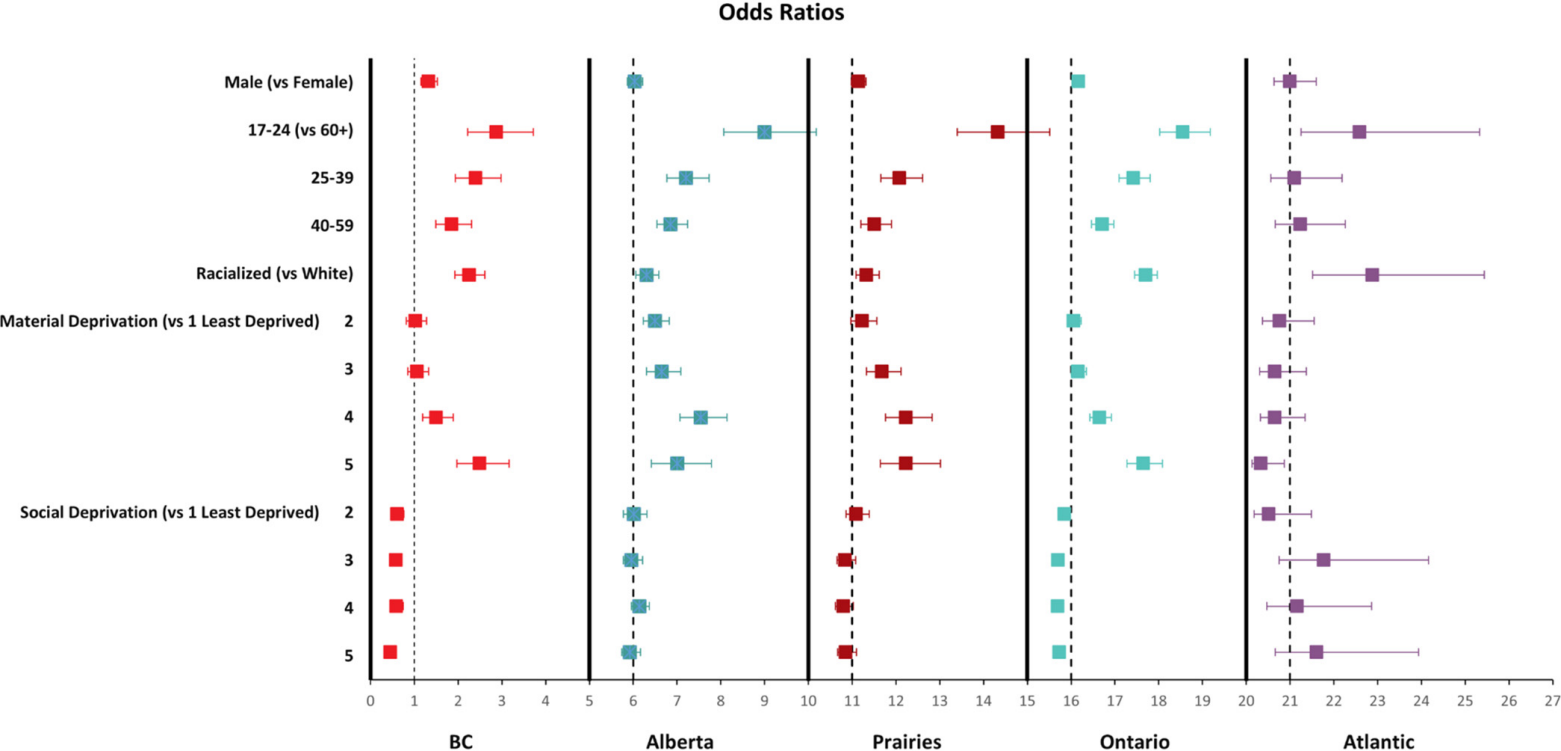
Regional multivariable odds ratios.

The proportions of nucleocapsid-positive (seropositive) donations for each variable by region are shown in Fig. 2. The multivariable models with all variables included are shown in Fig. 3 and in Table S3. Controlling for other variables, being racialized was significantly associated with seropositivity in Ontario, BC, and the Atlantic regions but not in Alberta or the Prairies. For both Alberta and the Prairies, with age and racialization and no other predictor variables, racialization was not significant (*P* = 0.5). No other variables were significantly associated with seropositivity in the Atlantic region. Although living in neighborhoods with greater material deprivation was a predictor of seropositivity in all regions except for the Atlantic, only quintile 5 in BC and quintiles 4 and 5 in Ontario were significant. Greater social deprivation was a predictor of lower seroprevalence in Ontario and BC but not elsewhere. Being male was a predictor in all regions except the Atlantic. Younger age was strongly associated with higher seroprevalence, and seroprevalence decreased with each progressively older age group. To test whether the inclusion of missing values could have altered the interpretation, multivariable models were fitted with the missing values excluded, and there was no substantive difference from the models with the missing values included. To test whether the fitting of the generalized estimating equation (GEE) model with the unique donor identifier adequately adjusted for the inclusion of more than one sample from some donors, a sensitivity analysis was conducted with one randomly selected donation from each donor, and all of the associations reported were unchanged.

## DISCUSSION

In this Canada-wide study, racialization was associated with higher SARS-CoV-2 seroprevalence in British Columbia and Ontario but not in Alberta and the Prairies. Living in a materially deprived neighborhood predicted higher seroprevalence. The COVID-19 pandemic was initially hailed as the great socioeconomic equalizer (25). To the contrary, it has exposed and amplified preexisting health inequity. Communicable disease is typically managed with a universal approach that assumes that everyone is affected by and benefits equally from the same types and intensities of interventions (26). Emerging analyses may suggest that interventions had less benefit for people at the lower end of the socioeconomic gradient, possibly even to their detriment (27, 28). There are calls to analyze and learn from the COVID-19 pandemic experience to facilitate a lasting change in health systems (29). Comparisons across regions with different population characteristics, health policies, and structures can provide insight into the underlying basis for inequity.

Worldwide, blood donors have contributed extensively to SARS-CoV-2 seroprevalence data (1, 3, 6, 30, 31), and these data are comparable with general population data (32, 33). Nevertheless, donors are a healthy adult population and are not representative of some segments, such as children, long-term care residents, men who have recently had sex with another man, those at high risk of many sexually transmitted bloodborne infections. and those living in remote and some rural areas (34). A fixed address and the ability to read and communicate in English (French in some areas) are essential for blood donation. The choice to donate blood also implies some degree of comfort with medical care. In contrast, research on health disparities tends to focus on the most marginalized communities, such as those in precarious housing, unable to afford personal protective equipment, with language barriers, and with a mistrust of medical care. The clear disparities associated with racialization and material deprivation in our donor population shed light on the pervasiveness of the socioeconomic gradient.

Donor seroprevalence by health region followed a similar trend to public health reported cases across all nine provinces and was similar to the PCR positive (case) percentage. However, there was one region in southern Alberta in which the donor seroprevalence was much higher than expected, based on reported cases. That region had a history of outbreaks, and higher seroprevalence was reported (19). Prior to the enhancement of public health testing capacity, the infection rate was estimated to be more than 4 times the observed rates from public health testing (35), but it was later reduced to about 2.5 times (36). Our results do not adjust for waning antibodies (37) but do suggest that fewer infections were missed by public health testing in 2021. We combined 12 months of seroprevalence data, over which there were 3 waves of the pandemic, fueled by new variants of concern (primarily Alpha and Delta) (38, 39). Due to the low seroprevalence in Atlantic Canada, we will focus this discussion on the regions from Ontario westward.

In Canada, health care is under provincial jurisdiction such that programs and facilities will vary across provinces. Health care is publicly funded, although some services, such as allied health and medications, are not. For these services, access is covered by a patchwork of private insurance and means tested public funding. Personal protective equipment, remote work where possible, and social distancing were advised by public health authorities over 2021, but policies varied by jurisdiction. Vaccination was deployed to high-risk groups and then from the oldest to the youngest age groups, as evidenced by vaccine antibodies in nearly all donors by mid-2021 (24, 40). The impact of time is not ruled out, however, as higher seroprevalence among younger donors, males, racialized groups, and those in more materially deprived and less socially deprived neighborhoods persisted over the full year (24).

Economic status is perhaps the most frequently mentioned pandemic social determinant of health (41, 42). Work from home was not available to about half of working Canadians, with a skew toward less availability in lower income households (43). SARS-CoV-2 was disproportionately represented in neighborhoods with higher density housing (44) and in those with more essential workers (those in health, food, manufacturing, and transportation) (17, 45). In our study, greater material deprivation predicted seroprevalence in all regions. Unlike Alberta and the Prairies, in Ontario and BC, the relationship differed by material deprivation, with similar seroprevalence in 60% of the population (quintiles 1 to 3) and higher odds ratios for the most deprived 40% (quintiles 4 and 5). The reasons for this distinct divide are unclear, but the material deprivation index is a neighborhood characteristic and hence may not always reflect individual characteristics. Social deprivation was associated with lower seroprevalence, primarily in BC and Ontario. It is possible that a clearer gradient between neighborhoods could be seen in these more densely populated provinces, whereas in less populous areas, the key determinants used to derive the index (living alone, divorced/widowed, single parent families) may not distinguish population density as well.

Racialization is associated with SARS-CoV-2 infection (14, 20, 46) and deaths (23) in Canada. It is a proxy for life situations associated with societal systematic and structural forces (28, 29). More overt discrimination was escalated for Chinese Canadians over the pandemic (28, 47, 48). In Alberta and the Prairies, racialization was not predictive of seroprevalence after age was considered. There are few publications from these regions, but associations between SARS-CoV-2 infection and living in racialized communities in Winnipeg (14) and Alberta (19) have been reported. A strength of our study is the use of donor-reported racialization status, as these data are not routinely collected by public health. Whereas the distribution of ethnicities in racialized donors overall reflects the general population, there could be variability at the community level, relative to the population, that we were unable to assess. Smaller numbers of donors in various nonwhite race-ethnicities necessitated considering racialized groups as a single entity but limited interpretation. In both Ontario and BC, racialized donors had higher seroprevalence after considering material deprivation and other variables. Presumably, other factors that were not measured in this study, such as front-line occupations and larger family sizes, influence the risk in racialized groups. These may be different across communities.

SARS-CoV-2 seroprevalence was higher in males than in females in all regions. There are several possible reasons for this. With the closures of schools, childcare may have fallen disproportionately to females, necessitating more time in the home and reducing social contacts. The risk for males in the workplace may be different from that of females (41, 49).

Younger age predicted SARS-CoV-2 seropositivity. Compliance with social distancing may have been perceived as less necessary with symptoms that were less severe, but it may also be less feasible due to reasons such as more employment in essential services and a greater reliance on public transport. The perceived risk of severe illness, combined with more work from home options, may have encouraged social distancing in older age groups. In Alberta and the Prairies, vaccination uptake was less than in other parts of Canada (https://health-infobase.canada.ca/covid-19/vaccination-coverage/archive/2021-12-24/), and the proportionately higher seropositivity among 17 to 24-year-olds was largely responsible for the overall higher seroprevalence. Possible factors include different social attitudes, the earlier removal of some restrictions, and the reduced feasibility of work from home.

In this large blood donor population across Canada, we observed higher SARS-CoV-2 seroprevalence among racialized individuals and those with material deprivation. These relationships persisted, even after accounting for confounding. These findings highlight the higher burden of infection related to factors that are traditionally associated with health disparities. Efforts are needed to address racial and socioeconomic disparities in COVID-19 infections and in other related health conditions.

## MATERIALS AND METHODS

### Study design and population.

All Canadian Blood Services donors must be at least 17 years of age and are required to answer screening questions to ensure that they are in good health and are not at risk of blood transmissible infections, and they must also have their temperatures checked. During the pandemic, additional deferrals were put in place to reduce the risk of SARS-CoV-2 infection for the donors and staff at the collection site. All donors were deferred from donating blood for 2 weeks if they had been in contact with someone who was infected or if they had had an infection (3 week deferral if hospitalized).

Canadian Blood Services collects donations from all provinces (except Quebec) but not in the Northern territories (Yukon, Northwest Territories, Nunavut). A combination of fixed and mobile sites collect blood in all larger cities. Mobile collection sites are available in many towns, generally every 3 months but sometimes less frequently. For some remote and rural areas, donation may only be possible when the donor travels to a town or city in which donations are collected. An extra ethylenediaminetetraacetic acid (EDTA) blood sample is routinely collected from all donors at the time of donation, in case any additional testing is required to qualify the blood product. About 80% of these samples are not used and were therefore available for SARS-CoV-2 serologic testing. Samples collected between January and December of 2021 were randomly selected for serologic testing on a monthly basis (24). Samples were collected during approximately the last 2 weeks of every month from January of 2021 to December of 2021, as shown in Fig. S1. A straight random sample was applied until June, after which the samples were stratified into age groups by region before being randomly selected to reduce the total monthly sample size while maintaining a sample that was representative of important population demographics (age and region). Donors could donate multiple times within this period, with a chance of being selected for the study. Thus, there were some donors who had more than one sample tested. The number of samples varied somewhat from month to month, with a total of 165,236 samples coming from 118,998 distinct donors over the year of 2021. This study was approved by the Canadian Blood Services Research Ethics Board.

### Serologic testing.

The EDTA plasma samples were aliquoted and frozen at −20°C or colder until the time of testing. All samples were tested at the Canadian Blood Services laboratory in Ottawa, Ontario, using a Roche Elecsys Anti-SARS-CoV-2 qualitative immunoassay (Roche Diagnostics International Ltd., Rotkreuz, Switzerland), which measured the total antibodies (including IgA, IgM, and IgG) to the SARS-CoV-2 recombinant protein nucleocapsid antigen (anti-N). No decrease in anti-N assay performance was identified with the early Omicron subvariants, such as BA.1 and BA.2, in the field (50), and, as the nucleocapsid sequences are identical for the more recent subvariants, such as BA.3 and BA.5, no decrease in performance is anticipated.

### Data management and statistical analysis.

Demographic variables were extracted from the Canadian Blood Services donor database and were added to the test data, including the donation date, Forward Sortation Area (FSA) from the residential postal code, sex, age, and self-reported race-ethnicity. Provinces were classified into geographical regions across Canada (British Columbia, Alberta, Prairies [included Saskatchewan and Manitoba], Ontario, and the Atlantic region [included New Brunswick, Nova Scotia, Prince Edward Island, and Newfoundland and Labrador]). Donors self-identified as either White, South Asian, Asian (East or other), Indigenous, Arabic, Black, Latin-American, or Other. Race-ethnicities were regrouped *a priori* as either “white” (the majority of donors) or “racialized groups”, as the proportions in the various nonwhite race-ethnicities were small. This differs from the Statistics Canada definition of visible minorities, which does not include Indigenous Peoples. Socioeconomic status was estimated by the Pampalon Material and Social Deprivation Indices (MSDI) (51, 52). Material deprivation is associated with an insecure job situation, an insufficient income, and a low education, whereas social deprivation refers to a fragile social network that is characterized by living alone, being a single parent, or being separated, divorced, or widowed. The MSDI were derived from the 2016 Statistics Canada census, aggregated from postal codes to the dissemination area (DA) level (the smallest geographic unit available in the Canadian census, considering 400 to 700 persons), and categorized into quintiles, from the least deprived [1] to the most deprived [5]. Donors were categorized into 4 different age groups: 17 to 24, 25 to 39, 40 to 59, and 60+ years old.

The numbers of PCR positive public health cases by health region (health unit) were accessed from publicly available data sets (http://www.bccdc.ca/health-info/diseases-conditions/covid-19/data, https://www.alberta.ca/stats/covid-19-alberta-statistics.htm#data-export, https://dashboard.saskatchewan.ca/health-wellness/covid-19/cases, https://www.gov.mb.ca/health/publichealth/surveillance/covid-19/index.html, https://data.ontario.ca/dataset/confirmed-positive-cases-of-covid-19-in-ontario). The numbers of population per health region were accessed from national census data (Catalogue number 82-402-x-2018001). The percentage of the general population that was SARS-CoV-2 PCR positive in 2021 was of the number of cases divided by the number of people. The seroprevalence values per health region with at least 1,000 donors tested were compared with the corresponding percentages of cases via the Pearson product-moment test.

To compare anti-N positivity by each of the above demographic groups, donations in the year of 2021 were analyzed. For the univariate comparisons, the data were weighted by ranking for the donor’s FSA, age group, and sex to make an inference to the general population, based on Statistics Canada data (catalogue number 98-400-X2016008). For FSAs with few donors, several adjacent FSAs were combined to include at least 500 donors. In cases where no FSA was recorded or if the donor did not reside in a province in which blood is collected (0.2% of samples), the weighting was based on the FSA of the blood center. The weighted data were adjusted for the sensitivity and specificity of the assay, as reported by the manufacturer, using the Rogan-Gladen equation (53). The seroprevalence was calculated as the number of positive samples divided by the total number of samples tested. The exact method was used to estimate 95% confidence intervals. The SARS-CoV-2 seroprevalence was stratified by region, sex, age group, racialization, and MSDI.

Logistic regression models were constructed for each geographic region with a unique donor identifier to identify all of the repeated measures in a GEE. Models were first constructed for each variable to assess univariate predictors of seropositivity. Then, a regression model was built for each region, including all variables. Age and sex were retained in all model iterations. Missing values for particular variables were included as a “missing” category. Because a primary objective was to evaluate the association of infection with racialization and material and social deprivation, these were evaluated individually and in combinations for each region, with the age group and sex included in the models. To test whether the inclusion of all samples from donors with more than one sample altered the interpretation, a sensitivity analysis was conducted, in which one sample from each donor was randomly selected.

All analyses were conducted using SAS (version 9.4, Cary, NC) and STATA/MP 17 (Statacorp. 2021. College Station, TX).

### Data availability.

An aggregate data set at the level permitted by the research ethics board is available upon request. Deidentified data at the individual level may be made available upon request from Canadian Blood Services (contact person: Sheila O’Brien), subject to internal review, privacy legislation, data sharing agreements, and research ethics approval.
