# Supplementary material for: Cross-Canada Variability in Blood Donor SARS-CoV-2 Seroprevalence by Social Determinants of Health
Source: Microbiol Spectr. 2023 Jan 10;11(1):e03356-22. doi: 10.1128/spectrum.03356-22 (PMC9927354; doi:10.1128/spectrum.03356-22)
Supplement: Supplemental file 1 — Supplemental material. Download spectrum.03356-22-s0001.pdf, PDF file, 0.4 MB [file spectrum.03356-22-s0001.pdf]

**Supplemental Table 1** Number and percentage of donors and donations by demographic groups

| | Donors (n=118,998) | | Donations (n=165,236) | |
|---|---|---|---|---|
| | **n** | **%** | **n** | **%** |
| **Sex** | | | | |
| *Female* | 55,838 | 46.92 | 69,085 | 41.81 |
| *Male* | 63,160 | 53.08 | 96,151 | 58.19 |
| **Age Group** | | | | |
| *17-24* | 13,073 | 10.99 | 15,499 | 9.38 |
| *25-39* | 34,217 | 28.75 | 44,335 | 26.83 |
| *40-59* | 42,006 | 35.30 | 59,480 | 36.00 |
| *60+* | 29,702 | 24.96 | 45,922 | 27.79 |
| **Province** | | | | |
| *BC* | 21,001 | 17.65 | 27,571 | 16.69 |
| *Alberta* | 23,035 | 19.36 | 34,636 | 20.96 |
| *Saskatchewan* | 6,366 | 5.35 | 8,885 | 5.38 |
| *Manitoba* | 6,985 | 5.87 | 9,450 | 5.72 |
| *Ontario* | 48,711 | 40.93 | 66,731 | 40.39 |
| *New Brunswick* | 3,884 | 3.26 | 5,567 | 3.37 |
| *Nova Scotia* | 5,489 | 4.61 | 7,379 | 4.47 |
| *PEI* | 1,138 | 0.96 | 1,759 | 1.06 |
| *Newfoundland* | 2,128 | 1.79 | 2,940 | 1.78 |
| *Missing* | 216 | 0.18 | 318 | 0.19 |
| **Region** | | | | |
| *BC* | 21,001 | 17.65 | 27,571 | 16.69 |
| *Alberta* | 23,035 | 19.36 | 34,636 | 20.96 |
| *Prairies* | 13,351 | 11.22 | 18,335 | 11.10 |
| *Ontario* | 48,711 | 40.93 | 66,731 | 40.39 |
| *Atlantic* | 12,639 | 10.62 | 17,645 | 10.68 |
| *Missing* | 261 | 0.22 | 318 | 0.19 |
| **Racialization** | | | | |
| *Racialized* | 20,230 | 17.00 | 25,915 | 15.68 |
| *White* | 88,348 | 74.24 | 124,541 | 75.37 |
| *Missing* | 10,420 | 8.76 | 14,780 | 8.94 |
| **Social Deprivation** | | | | |
| *1 (least deprived)* | 22,033 | 18.52 | 30,716 | 18.59 |
| *2* | 22,395 | 18.82 | 31,345 | 18.97 |
| *3* | 21,213 | 17.83 | 29,571 | 17.90 |
| *4* | 19,547 | 16.43 | 27,175 | 16.45 |
| *5 (most deprived)* | 19,993 | 16.80 | 27,691 | 16.76 |
| *Missing* | 13,817 | 11.61 | 18,738 | 11.34 |
| **Material Deprivation** | | | | |
| *1 (least deprived)* | 30,989 | 26.04 | 44,202 | 26.75 |
| *2* | 25,537 | 21.46 | 35,678 | 21.59 |
| *3* | 21,610 | 18.16 | 29,837 | 18.06 |
| *4* | 17,043 | 14.32 | 23,431 | 14.18 |
| *5 (most deprived)* | 10,002 | 8.41 | 13,350 | 8.08 |
| *Missing* | 13,817 | 11.61 | 18,738 | 11.34 |

**Supplemental Table 2** Percentage of positive donations by province

| Province | Tested | Positive | |
|---|---|---|---|
| | n | n | % |
| BC | 27,571 | 1,005 | 3.65 |
| Alberta | 34,636 | 2,389 | 6.90 |
| Saskatchewan | 8,885 | 423 | 4.76 |
| Manitoba | 9,450 | 571 | 6.04 |
| Ontario | 66,731 | 2,453 | 3.68 |
| New Brunswick | 5,567 | 24 | 0.43 |
| Nova Scotia | 7,379 | 45 | 0.61 |
| Prince Edward Island | 1,759 | 3 | 0.17 |
| Newfoundland | 2,940 | 14 | 0.48 |

**Supplemental Table 3** Regional multivariable odds ratios

| | BC (n=27,571) | | Alberta (n=34,636) | | Prairies (n=18,335) | | Ontario (n=66,731) | | Atlantic (n=17,645) | |
|---|---|---|---|---|---|---|---|---|---|---|
| | **OR (95% CI)** | **p-value** | **OR (95% CI)** | **p-value** | **OR (95% CI)** | **p-value** | **OR (95% CI)** | **p-value** | **OR (95% CI)** | **p-value** |
| **Variables** | | | | | | | | | | |
| ***Male (vs Female)*** | 1.30 (1.12, 1.50) | 0.0005 | 1.15 (1.04, 1.27) | 0.0061 | 1.23 (1.06, 1.43) | 0.0069 | 1.17 (1.07, 1.29) | 0.0009 | 1.04 (0.64, 1.68) | 0.8875 |
| ***Age Group (vs 60+)*** | | | | | | | | | | |
| *17-24* | 2.40 (1.83, 3.14) | <0.0001 | 3.96 (3.31, 4.73) | <0.0001 | 4.49 (3.50, 5.77) | <0.0001 | 2.89 (2.43, 3.42) | <0.0001 | 2.11 (0.99, 4.49) | 0.0530 |
| *25-39* | 2.19 (1.75, 2.75) | <0.0001 | 2.22 (1.88, 2.62) | <0.0001 | 2.18 (1.72, 2.77) | <0.0001 | 2.01 (1.73, 2.34) | <0.0001 | 0.86 (0.43, 1.73) | 0.6742 |
| *40-59* | 1.72 (1.37, 2.15) | <0.0001 | 1.84 (1.57, 2.17) | <0.0001 | 1.56 (1.23, 1.97) | 0.0002 | 1.55 (1.33, 1.80) | <0.0001 | 1.15 (0.60, 2.18) | 0.6804 |
| ***Racialized (vs White)*** | 1.68 (1.43, 1.97) | <0.0001 | 1.07 (0.94, 1.22) | 0.2978 | 0.99 (0.80, 1.21) | 0.8962 | 2.06 (1.86, 2.28) | <0.0001 | 2.66 (1.43, 4.95) | 0.0020 |
| ***Material Deprivation (vs 1 Least Deprived)*** | | | | | | | | | | |
| *2* | 0.96 (0.76, 1.21) | 0.7237 | 1.47 (1.28, 1.70) | <0.0001 | 1.22 (0.96, 1.56) | 0.1016 | 1.05 (0.91, 1.22) | 0.4979 | 0.78 (0.38, 1.62) | 0.5022 |
| *3* | 0.94 (0.75, 1.19) | 0.6119 | 1.62 (1.39, 1.89) | <0.0001 | 1.65 (1.30, 2.09) | <0.0001 | 1.13 (0.98, 1.31) | 0.0989 | 0.70 (0.32, 1.50) | 0.3564 |
| *4* | 1.24 (0.98, 1.57) | 0.0673 | 2.44 (2.08, 2.85) | <0.0001 | 2.17 (1.71, 2.76) | <0.0001 | 1.55 (1.34, 1.80) | <0.0001 | 0.71 (0.34, 1.46) | 0.3506 |
| *5 (Most Deprived)* | 1.69 (1.32, 2.16) | <0.0001 | 1.86 (1.46, 2.36) | <0.0001 | 2.12 (1.54, 2.91) | <0.0001 | 2.20 (1.89, 2.57) | <0.0001 | 0.37 (0.14, 0.97) | 0.0427 |
| ***Social Deprivation (vs 1 Least Deprived)*** | | | | | | | | | | |
| *2* | 0.65 (0.52, 0.81) | 0.0001 | 1.00 (0.85, 1.19) | 0.9674 | 1.20 (0.94, 1.53) | 0.1490 | 1.00 (0.87, 1.14) | 0.9432 | 0.55 (0.19, 1.59) | 0.2714 |
| *3* | 0.65 (0.52, 0.82) | 0.0002 | 0.93 (0.78, 1.10) | 0.3760 | 0.87 (0.67, 1.13) | 0.2998 | 0.77 (0.67, 0.90) | 0.0006 | 1.85 (0.79, 4.36) | 0.1591 |
| *4* | 0.66 (0.52, 0.83) | 0.0005 | 1.05 (0.89, 1.24) | 0.5478 | 0.80 (0.62, 1.03) | 0.0846 | 0.70 (0.60, 0.81) | <0.0001 | 1.15 (0.47, 2.81) | 0.7624 |
| *5 (Most Deprived)* | 0.50 (0.40, 0.63) | <0.0001 | 0.93 (0.78, 1.10) | 0.3875 | 0.82 (0.63, 1.05) | 0.1204 | 0.67 (0.58, 0.78) | <0.0001 | 1.66 (0.68, 4.07) | 0.2688 |

Supplemental Figure 1 Numbers of samples tested per month

| 2021 | | | | | | | | | | | |
|---|---|---|---|---|---|---|---|---|---|---|---|
| January | February | March | April | May | June | July | August | September | October | November | December |
| 34,280 | 34 | 16,949 | 17,027 | 17,042 | 17,003 | 8,465 | 9,246 | 9,446 | 9,653 | 9,124 | 16,967 |

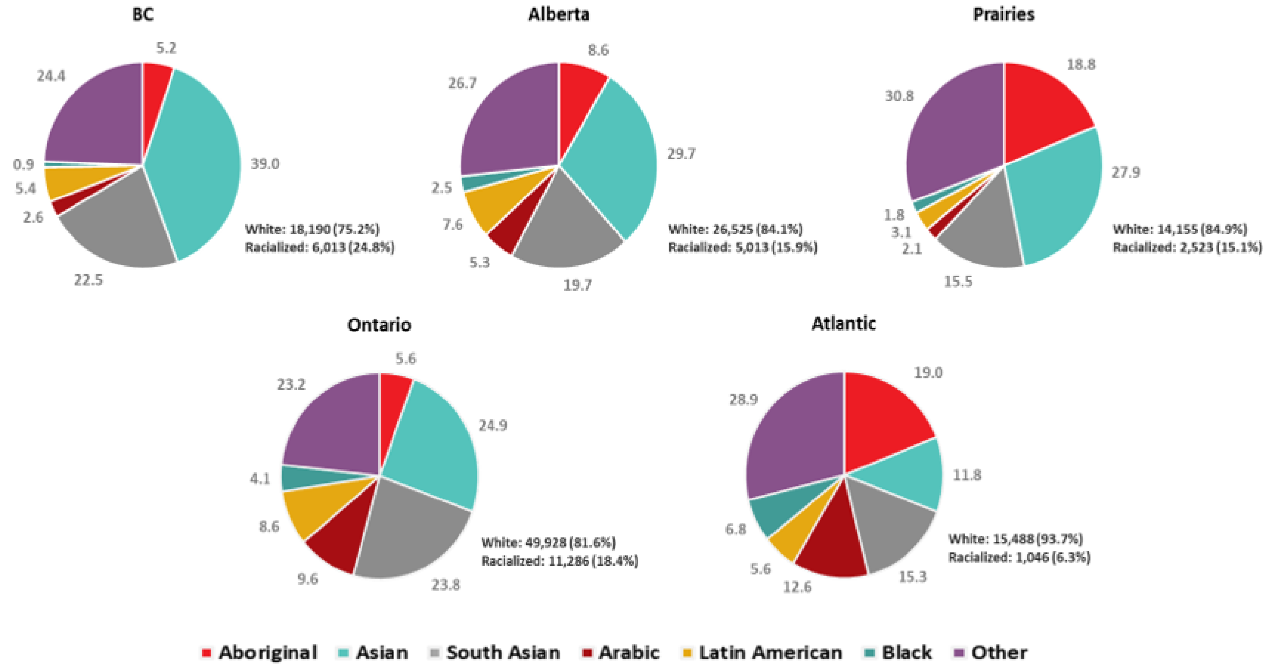


**Supplemental Figure 2** Pie charts of regional racialized donors by self selected ethnicities
